# Right trisectionectomy for liver metastasis of granulosa cell tumor: a case report and literature review

**DOI:** 10.1186/s40792-020-00880-3

**Published:** 2020-06-03

**Authors:** Itsuki Koganezawa, Koichi Tomita, Masashi Nakagawa, Yosuke Ozawa, Toshimichi Kobayashi, Toru Sano, Rina Tsutsui, Naokazu Chiba, Akira Okimura, Munehide Nakatsugawa, Hiroshi Hirano, Shigeyuki Kawachi

**Affiliations:** 1 Department of Digestive and Transplantation Surgery, Tokyo Medical University Hachioji Medical Center, 1163 Tatemachi, Hachiojishi, Tokyo, 193-0998 Japan; 2 Department of Diagnostic Pathology, Tokyo Medical University Hachioji Medical Center, 1163 Tatemachi, Hachiojishi, Tokyo, 193-0998 Japan

**Keywords:** Granulosa cell tumor, Liver metastasis, Late recurrence, Right trisectionectomy

## Abstract

**Background:**

Granulosa cell tumor (GCT) is a type of ovarian sex cord-stromal tumor with low-grade malignancy, which can recur long after primary resection. All reports on GCTs in the liver describe cases of metastases, while there are no previous reports of primary GCTs originating from the liver. We report a case of GCT, with recurrence of liver metastasis long after ovariectomy, which was subsequently resected by a right trisectionectomy.

**Case presentation:**

A 76-year-old woman presented with a history of surgical resection of an ovarian tumor performed 30 years previously; no details of the tumor were available. When she was 68 years old, an abdominal ultrasound revealed a small liver mass, which was diagnosed as a hepatic hemangioma with slow growth. Outpatient follow-up was discontinued for 5 years, and the patient was not examined again until the age of 76 years. At this point, the tumor had substantially increased in size, and surgical resection was required owing to suspicion of malignancy. The patient was then referred to our hospital. Contrast-enhanced computed tomography (CT) showed a large tumor, approximately 18 cm in size, occupying the right lobe and medial section of the liver. After percutaneous transhepatic portal vein embolization, a right trisectionectomy was performed. The histopathological findings of the resected specimen showed that the tumor cells had “coffee bean-like” nuclear grooves, which are characteristic of a GCT. Acidophilic non-structural Call-Exner bodies were also observed. Inhibin-α, CD99, and CD56 markers of sex cord-stromal tumors were detected on immunohistological examination; all pathology suggested a GCT. We considered the tumor to be a liver metastasis of a previous ovarian GCT that was resected 30 years prior by ovariectomy. There was no recurrence for > 15 months after the hepatectomy.

**Conclusions:**

We report a case of a GCT in the liver, which was identified to be a liver metastasis. Right trisectionectomy was subsequently performed for tumor resection. Clinicians should be aware that ovarian GCTs may recur in the liver, and that GCT recurrence may occur long after ovariectomy of the primary ovarian GCT.

## Background

According to the World Health Organization’s histological classification of ovarian tumors (2014), a granulosa cell tumor (GCT) is a type of sex cord-stromal tumor with low-grade malignancy [1]. These sex cord-stromal cell tumors comprise of pure sex cord tumors and pure stromal tumors. The GCT is categorized as a pure stromal tumor, accounting for 2–5% of ovarian tumors and 70% of sex cord-stromal tumors. The GCT is a relatively rare ovarian tumor classified as either an adult GCT, with an incidence of 95%, or a juvenile GCT, with an incidence of 5% [2]. A GCT is usually treated by ovariectomy and has a relatively good prognosis; however, late recurrence can arise. GCT progression often manifests with pelvic recurrence or peritoneal dissemination; whereas, liver metastasis is rarely noted (5–6% of cases) [3]. All reports on the occurrence of GCTs in the liver describe cases of metastases [3], but none of these reports have described a GCT originating from the liver. Herein, we report a case of a GCT with a recurrence of liver metastasis long after ovariectomy, which was subsequently treated by right trisectionectomy of the liver.

## Case presentation

A 76-year-old woman presented to our hospital with a history of ovariectomy for an ovarian tumor that was performed 30 years prior. Additional details related to the tumor were unavailable. When she was 68 years old (22 years after the ovariectomy), a local doctor noted a gallbladder polyp and a 3-cm liver mass when performing an abdominal ultrasound. The tumor was considered to be a hepatic hemangioma showing slow growth; however, her outpatient follow-up was discontinued for the next 5 years. A repeat imaging examination revealed that the liver tumor had increased to 17 cm in size. Consequently, she was referred to our hospital for detailed examination and surgical resection.

Almost all blood tests showed normal results, except for the detection of mild anemia, elevated biliary enzyme levels, and an inflammatory response. The abnormal values were as follows: hemoglobin, 10.3 g/dl; albumin, 3.0 g/dl; alkaline phosphatase, 557 U/l; lactate dehydrogenase, 250 U/l; and C-reactive protein, 1.21 mg/dl. The tumor markers, carcinoembryonic antigen, carbohydrate antigen 19-9, α-fetoprotein (AFP), protein induced by the absence of vitamin K or antagonist II, and AFP-L3, were all negative. Further blood tests showed no evidence of viral hepatitis, testing negative for hepatitis B surface antigens, hepatitis B surface antibodies, and hepatitis C virus antibodies.

Abdominal ultrasonography performed previously revealed that the tumor was spreading around the right lobe of the liver and appeared to be a combination of two masses. High and low echoes and blood flow signals were detected inside the tumor.

**Fig. 1:**
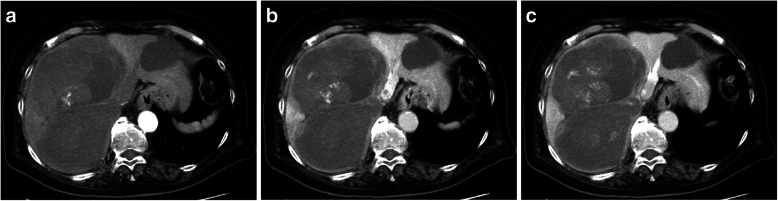
Computed tomography images. A contrast-enhanced dynamic computed tomography image of a tumor 18 cm in diameter in the right hepatic lobe. **a** Arterial phase, **b** portal vein phase, and **c** equilibrium phase

The abdominal contrast-enhanced dynamic computed tomography (CT) image revealed a maximum tumor diameter of ~ 18 cm in the right lobe and medial section of the liver (Fig. 1a–c). The tumor had an uneven and prolonged contrast effect during the early and equilibrium phases of imaging, respectively.

**Fig. 2:**
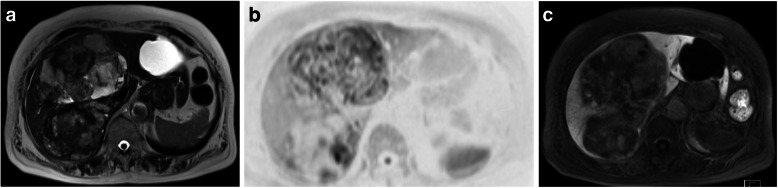
Magnetic resonance imaging findings. **a** Magnetic resonance image showing a tumor with mixed high- and low-intensity signals on T2-weighted imaging (HASTE). **b** Diffusion-weighted image showing a high signal intensity of the tumor. **c** The dynamic 15-mm hepatobiliary phase image of the prolongation of the internal contrast effect

Gadolinium ethoxybenzyl diethylenetriaminepentaacetic acid-enhanced magnetic resonance imaging (MRI) showed a tumor with mixed high- and low-signal intensities on T2-weighted imaging (Fig. 2a). The tumor had a mosaic-like internal environment, suggesting bleeding and necrosis. Diffusion-weighted images revealed a high-signal intensity (Fig. 2b). The dynamic 15-min hepatobiliary phase image showed prolongation of the internal contrast effect (Fig. 2c). Positron emission tomography findings did not reveal any significant fluorodeoxyglucose accumulation in the hepatic tumor or other parts of the body. Thus, the tumor was suspected to be a liver hemangiosarcoma. Malignancy was possible since the tumor had grown over time, so we decided to perform a surgical resection, and right trisectionectomy was planned as the tumor occupied the right lobe and medial section of the liver.

The preoperative evaluation of the liver function showed an indocyanine green 15-min retention rate (ICG-R15) of 13%, and a Child-Pugh score of 5 points. Since our estimation of the remnant liver function using ^99m^Tc-GSA scintigraphy [4] was insufficient, percutaneous transhepatic portal vein embolization (PTPE) was performed to improve the function of the remnant liver. After PTPE, the future liver remnant volume increased from 501 to 636 mL, and the ICG-R15 did not change. According to the ^99m^Tc-GSA scintigraphy, the LU15 value—which should be more than 13 to prevent post-hepatectomy liver failure—improved from 12.5 to 13.2. Two months after the PTPE, it was decided that surgery would be performed.

**Fig. 3:**
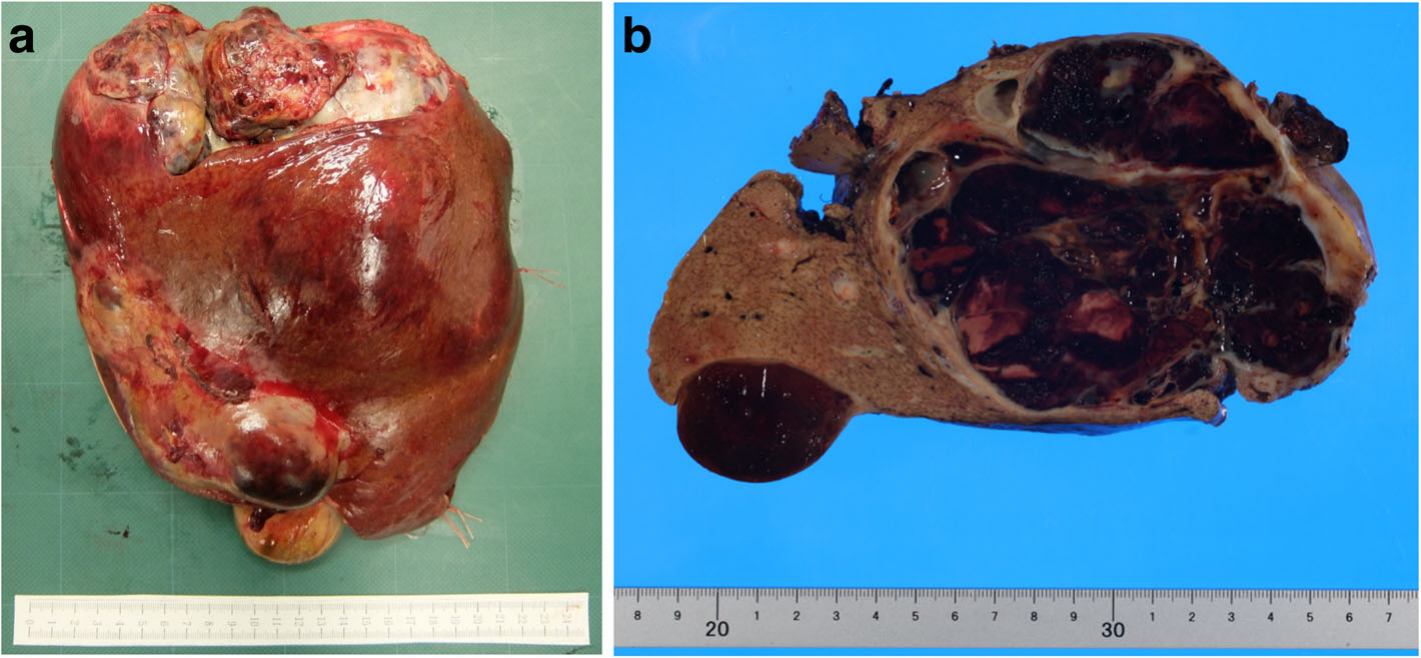
Surgical specimen. Macroscopically, the tumor has a nodule with a black-brown and yellowish-white composition with septum formation on the cut surface

**Fig. 4:**
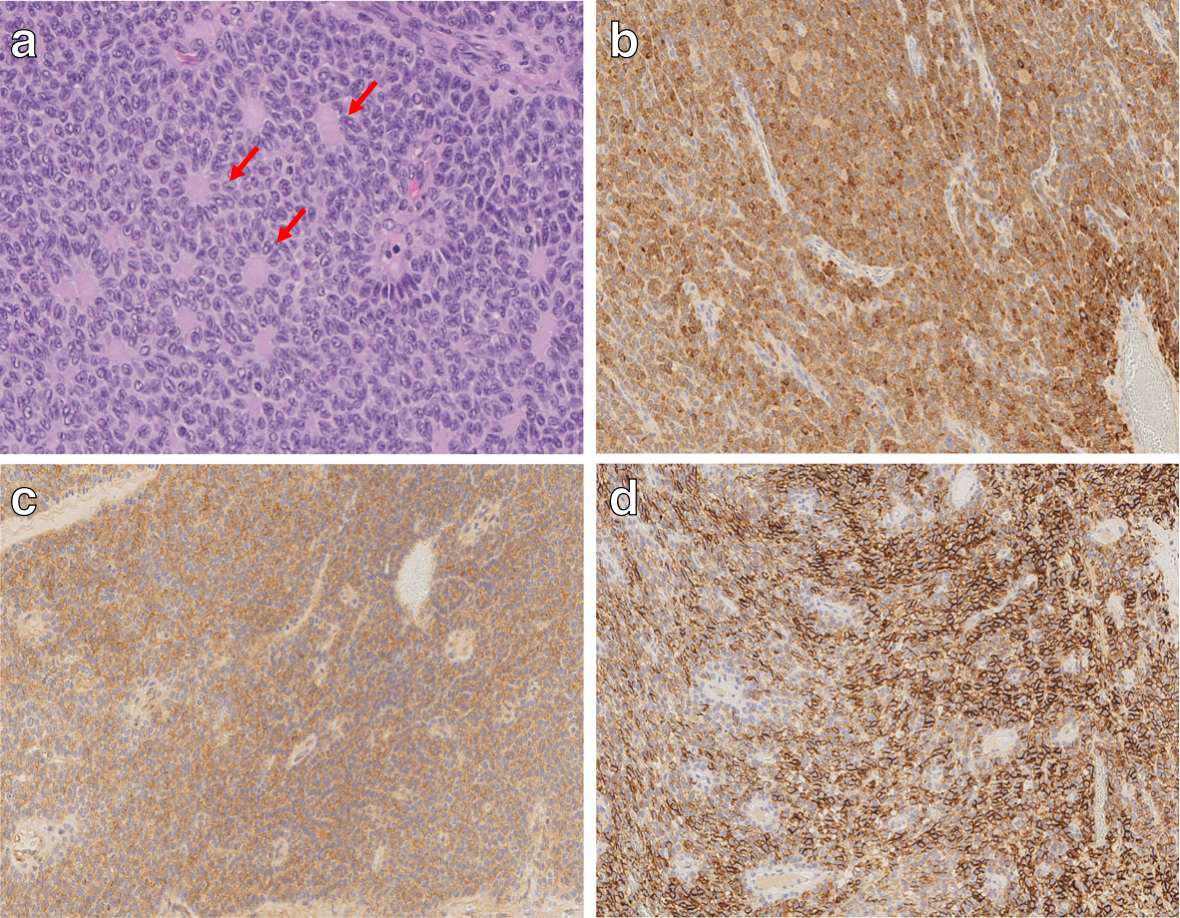
Immunohistochemical results. **a** Hematoxylin and eosin-stained image shows an acidophilic non-structural substance (Call-Exner body) in the center of the rosette-like structure (red arrow) and a “coffee bean-like” nuclear groove. Immunostaining is also performed for **b** inhibin-α (× 20), **c** CD99 (× 20), and **d** CD56 (× 20)

The surgical findings indicated that part of the tumor had invaded the diaphragm and thoracic cavity. Hence, a thoracolaparotomy was performed to remove the tumor by opening the chest through the sixth intercostal space and resecting the tumor that had invaded the diaphragm. We then performed a right hepatic trisectionectomy. The operative time was 306 min, and the estimated blood loss was 1475 ml. The size of the resected specimen was 22 × 17 × 8 cm (Fig. 3), and we observed a 10 × 9 cm nodule of black-brown and yellowish-white composition with septum formation (Fig. 3). The hematoxylin and eosin (HE)-stained specimens showed tumor cells growing in sheets (diffuse pattern) or cords (trabecular pattern), while rosettes (microfollicular pattern; Fig. 4a) were also visible. The nuclei of the tumor cells were relatively uniform and showed “coffee bean-like” nuclear grooves—characteristic of GCTs (Fig. 4a). Additionally, an acidophilic non-structural substance (Call-Exner body) was present in the center of the rosette-like structure (Fig. 4a), which is another hallmark characteristic of ~ 30–50% of GCT cases. Pathological findings were suggestive of a GCT; therefore, we performed additional immunostaining (results are in Table 1). The following markers were identified in the specimen: inhibin-α (Fig. 4b), CD99 (Fig. 4c), and CD56 (Fig. 4d), which were similar to that noted in sex cord-stromal cells [5, 6]. These findings helped to rule out germ cell tumors, which indicate the presence of other markers that were not detected in our evaluation, including the Sal-like protein, AFP, and β-human chorionic gonadotropin. Based on these findings, we diagnosed the tumor as a GCT. This tumor may have been a liver metastasis of the ovarian tumor recognized 22 years after the ovariectomy, and the liver metastasis was resected 8 years after its discovery. There was no recurrence for > 15 months after the hepatectomy.

**Table 1:** Markers for immunostaining

	Marker	Characteristic
Positive	Inhibin-α	Inhibin-α is present in most sex cord-stromal cells, and the sensitivity and specificity of inhibin-α for detecting sex cord-stromal tumors is 56% and 96%, respectively [5].
	CD99	CD99 is detected on the cell surface of Ewing’s sarcoma tumors, ependyma, and granulosa cell tumors.
	CD56	Neuroendocrine tumors are positive for CD56, and CD56 is a marker for granulosa cell tumors. CD56 is useful in distinguishing between granulosa cell tumors and normal ovarian follicles or endometrioid adenocarcinomas [6].
Negative	SALL4	SALL4 is a marker for germ cell tumors.
	AFP	AFP is a fetal protein and a marker for hepatocellular carcinoma, hepatoblastoma, and germ cell tumors.
	β-HCG	Villous tumors are positive for HCG.

*AFP* α-fetoprotein, *β-HCG* β-human chorionic gonadotropin, *SALL4* Sal-like protein 4

## Conclusions

We report a case of a GCT with liver metastasis detected 22 years after an ovariectomy which was successfully treated by right trisectionectomy. Preoperatively, a tumor biopsy was not performed, and we could not achieve an accurate diagnosis. The postoperative pathological findings suggested a GCT. The HE-stained specimens showed tumor cells with “coffee bean-like” nuclear grooves and Call-Exner bodies, which are typical characteristics of GCTs. In our case, a germ cell tumor was considered as a differential diagnosis. However, the immunohistochemical results indicated that markers of sex cord-stromal cells were detected, while markers of germ cell tumors were not. Therefore, the possibility of a germ cell tumor was ruled out. The pathological findings from HE stains and immunohistochemical analysis were consistent with the findings of a GCT. We noted that a GCT originating from the liver had not been previously reported and concluded that this GCT in the liver was a metastatic lesion of an ovarian tumor that was resected 30 years prior.

In total, 17 cases of hepatectomy for GCT liver metastasis, including our case, have been reported since the first description by Garcia et al. in 1996 (Table 2). Most cases had a long time interval between primary diagnosis and hepatectomy, which was similar to our case. In our patient, the GCT presented with liver metastasis 22 years after ovariectomy, which is expected considering that the GCT is known to have a late recurrence. The recurrence rate of GCTs is 32%, and recurrence-free survival for GCTs is 8.4 (6.8–9.9) years [15]. Moreover, tumors often recur after 10 years, with late recurrence of up to 40 years noted in some cases [16]. Therefore, a relatively long-term follow-up is required to monitor the treatment outcomes of GCTs.

**Table 2:** Previous reports of patients with GCT liver metastasis who underwent surgical resection

Authors	Year of publication	No. of cases	Time from primary diagnosis to hepatectomy (years)	Time from liver metastasis to hepatectomy (years)	Surgical procedure	Combined resection of other organs	Recurrence
Rodriguez Garcia JI, et al. [7]	1996	1	6	5	Right hepatectomy	No	No
Crew et al. [8]	2005	1	12	10	Partial	Yes	Yes
Madhuri et al. [9]	2010	3	6–17	0–2.7	Partial	Yes	No
Chua et al. [10]	2011	2	6–12	0–14.3	Partial, right hepatectomy	Yes	1 Yes, 1 no
Andreou et al. [11]	2012	5	Unknown	Unknown	Unknown	Unknown	Unknown
Fujita et al. [12]	2015	1	25	Unknown	Right hepatectomy	Yes	Yes
Yu et al. [13]	2015	1	27	Unknown	Bisegmentectomy	No	No
Antony et al. [14]	2017	2	5, 3	4, 2	Partial, right hepatectomy	Yes	No
Present case	2020	1	30	> 8	Right trisectionectomy	No	No

*GCT* granulosa cell tumor

Regarding the tumor growth speed, previous reports have described a relatively long interval from the diagnosis of liver metastasis to hepatectomy (Table 2). In our case, the liver metastasis had slow growth before the local doctor first detected it; however, the growth rate later increased. To our knowledge, no previous study has reported the acceleration of tumor growth over time. A GCT is classified as a low-grade malignant tumor that rarely grows rapidly; however, Inada et al. reported a juvenile GCT case that showed a rapid 12 cm increase in approximately 1 year [17]. In our case, we hypothesized that the nature of the tumor, and the speed of tumor growth, may have changed over time.

There is no standard-of-care or clinical practice guideline recommended for the management of recurrent GCTs; however, various approaches, including surgical resection, chemotherapy, and radiation therapy are used in the clinical setting treating their recurrence GCTs [18–20]. Moreover, previous reports have indicated that surgical resection to eradicate the residual disease can improve a patient’s postoperative quality of life and recurrence-free survival [2, 14, 21]. In our case, liver metastasis was assumed to be the only form of GCT recurrence, with no further signs of metastases. Therefore, we performed a right trisectionectomy, which is the most extensive hepatectomy reported thus far (Table 2).

Moreover, this metastatic tumor recurred after a prolong duration of 22 years. It was thought that surgical resection could achieve a disease-free status, and long-term survival was expected. We have followed up with this patient for > 15 months since the hepatectomy, and no recurrence or metastasis has been detected. Conversely, the possibility of another recurrence cannot be disregarded as GCTs are known for their repeated recurrence. Accordingly, we believe that careful follow-up is vital for this patient.

We report a case of a GCT in the liver, which was considered to be a liver metastasis and was subsequently resected by performing right trisectionectomy. Clinicians should be aware that ovarian GCTs may recur in the liver, and that this recurrence may occur many years after performing the primary ovarian GCT’s ovariectomy.

## Data Availability

All data generated or analyzed during this study were included in this published article.

## Abbreviations

GCT: Granulosa cell tumor
CT: Computed tomography
MRI: Magnetic resonance imaging
AFP: α-Fetoprotein
ICG-R15: Indocyanine green 15-min retention rate
PTPE: Percutaneous transhepatic portal vein embolization
HE: Hematoxylin and eosin
